# Supplementary material for: Genetic barriers more than environmental associations explain Serratia marcescens population structure
Source: Commun Biol. 2024 Apr 17;7:468. doi: 10.1038/s42003-024-06069-w (PMC11023947; doi:10.1038/s42003-024-06069-w)
Supplement: Supplementary file 1 — Supplementary Information [file 42003_2024_6069_MOESM1_ESM.pdf]

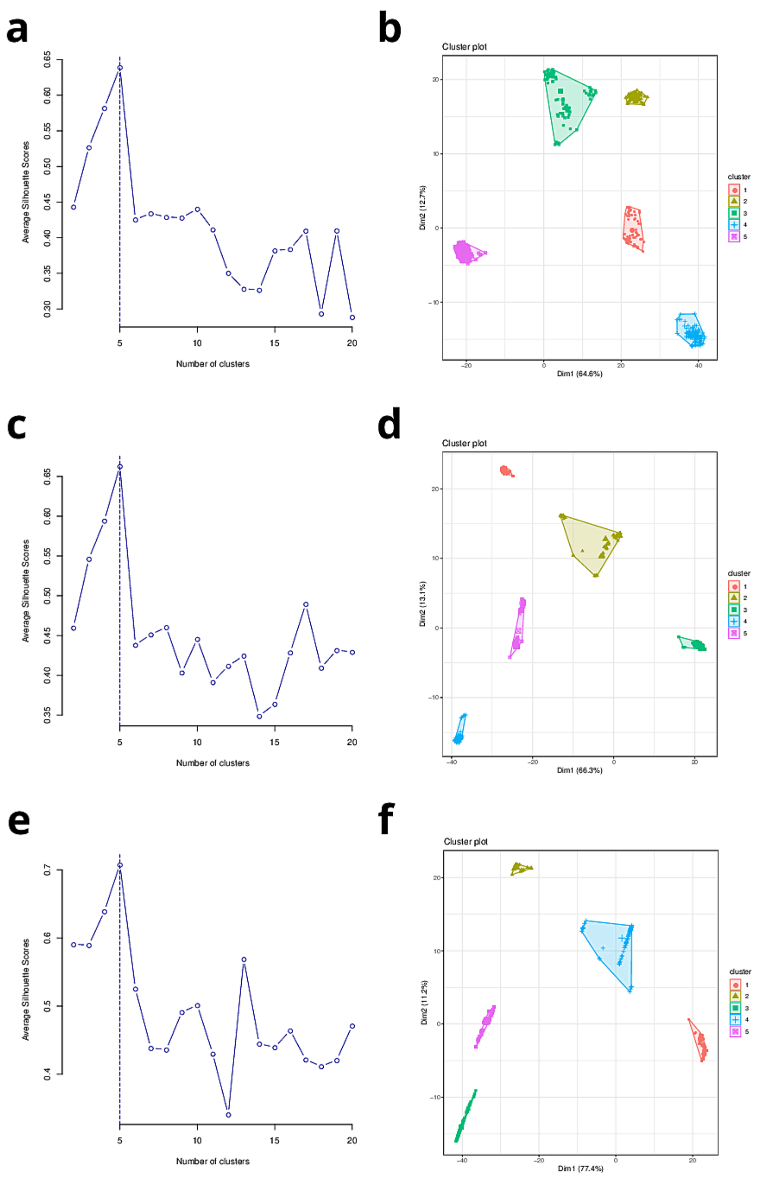

Figure S1. K-means clustering on core Single Nucleotide Polymorphisms distances.

a), c) and e) Silhouette plot of the number of clusters on the basis of Mash distances, core Single Nucleotide Polymorphisms (coreSNP) distances and patristic distances between *S. marcescens* strains. The number of clusters with the highest score was chosen as the optimal number of clusters to fit the data in. b), d) and f) Principal Coordinate Analysis (PCoA) performed on Mash distances, SNP distances and patristic distances, coloured on the basis of the clusters identified by the K-means algorithm.

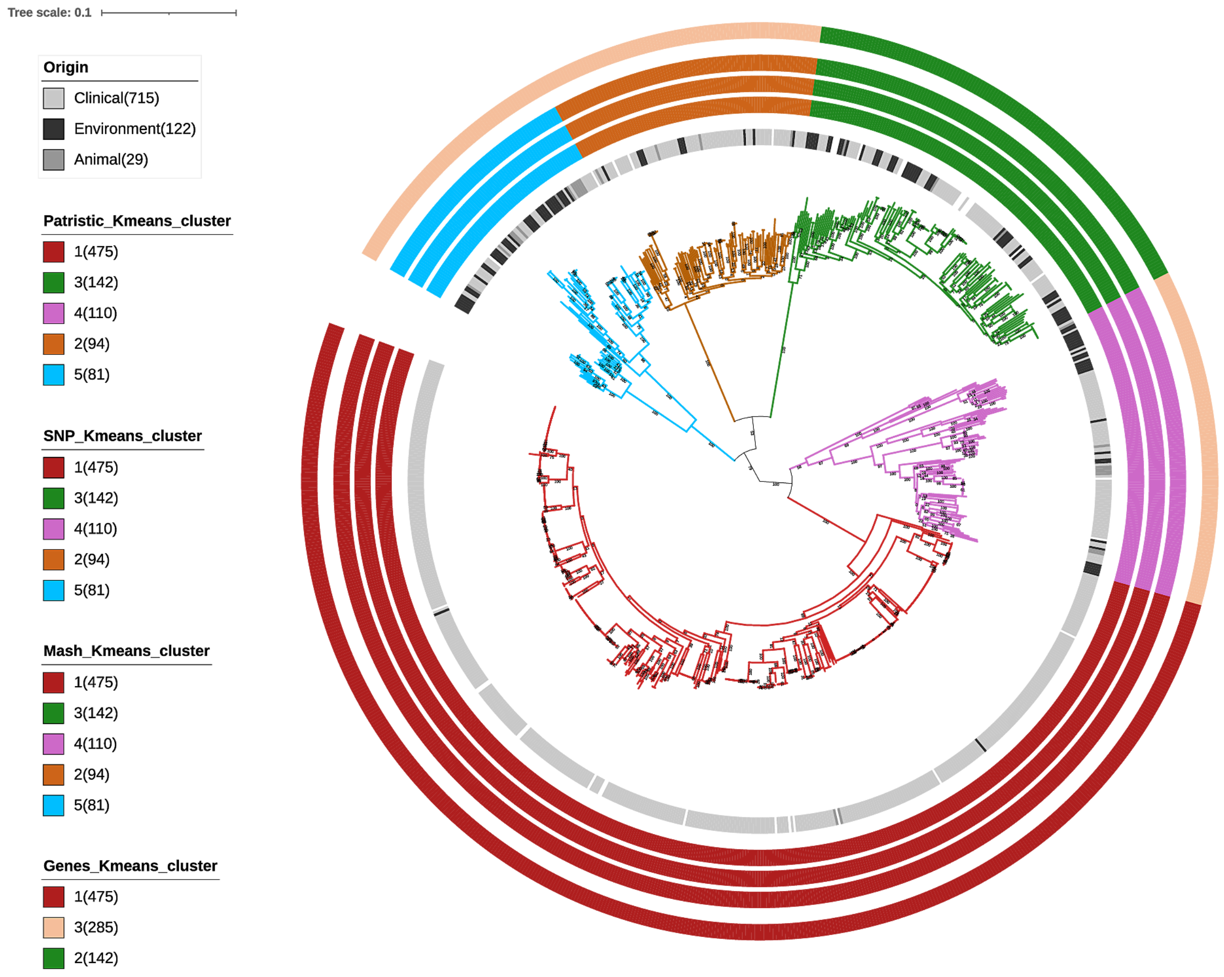

Figure S2. Clusters mapped on the phylogenetic tree.

NP-based Maximum Likelihood (ML) phylogenetic tree of the 902 *Serratia marcescens* strains of the Global genomic dataset. The tree branches' colours and the inner circle around the tree indicate the five clusters coherently and independently determined applying K-means clustering on patristic distances, coreSNP distances and Mash distances. The inner circle around the tree indicates the strain isolation source (blank if not traceable from the metadata). The three circles in the middle indicate the clusters inferred from, respectively, patristic distances, coreSNP distances and Mash distances. The outer circle indicate the three clusters inferred from the gene presence absence matrix. Bootstrap values are shown on the tree nodes.

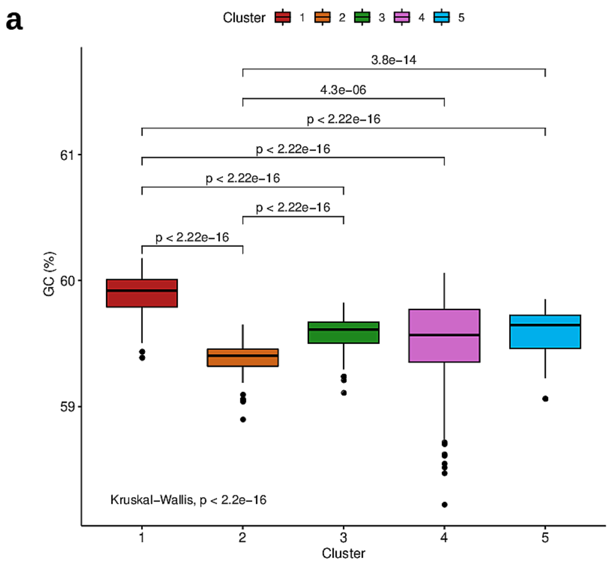

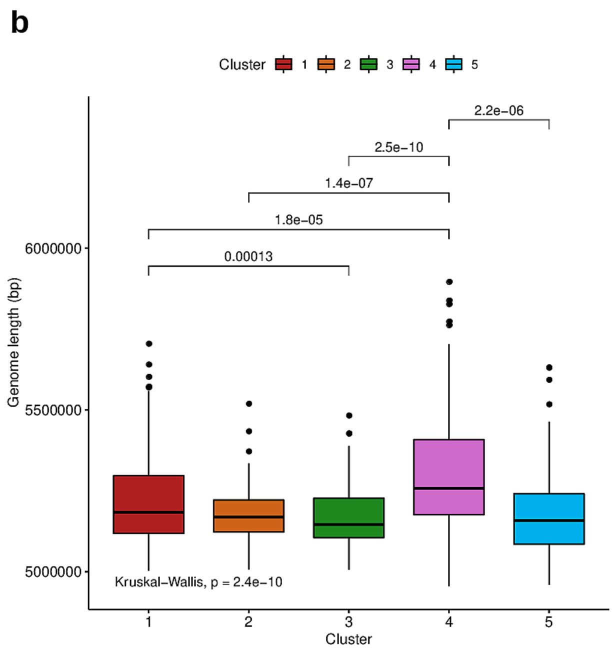

Figure S3. GC content and genome length in *S. marcescens* clusters.

a) Boxplot showing the percentage of GC content in the genomes of each cluster. The p-value of the Kruskal-Wallis test, performed to test the variance between groups, is shown on the bottom left. The groups with significant pairwise differences (Mann-Whitney U test) are connected and the p-value is written on top.

b) Boxplot showing the genome sizes of each cluster. Kruskal-Wallis test and Mann-Whitneu U test are reported as above.

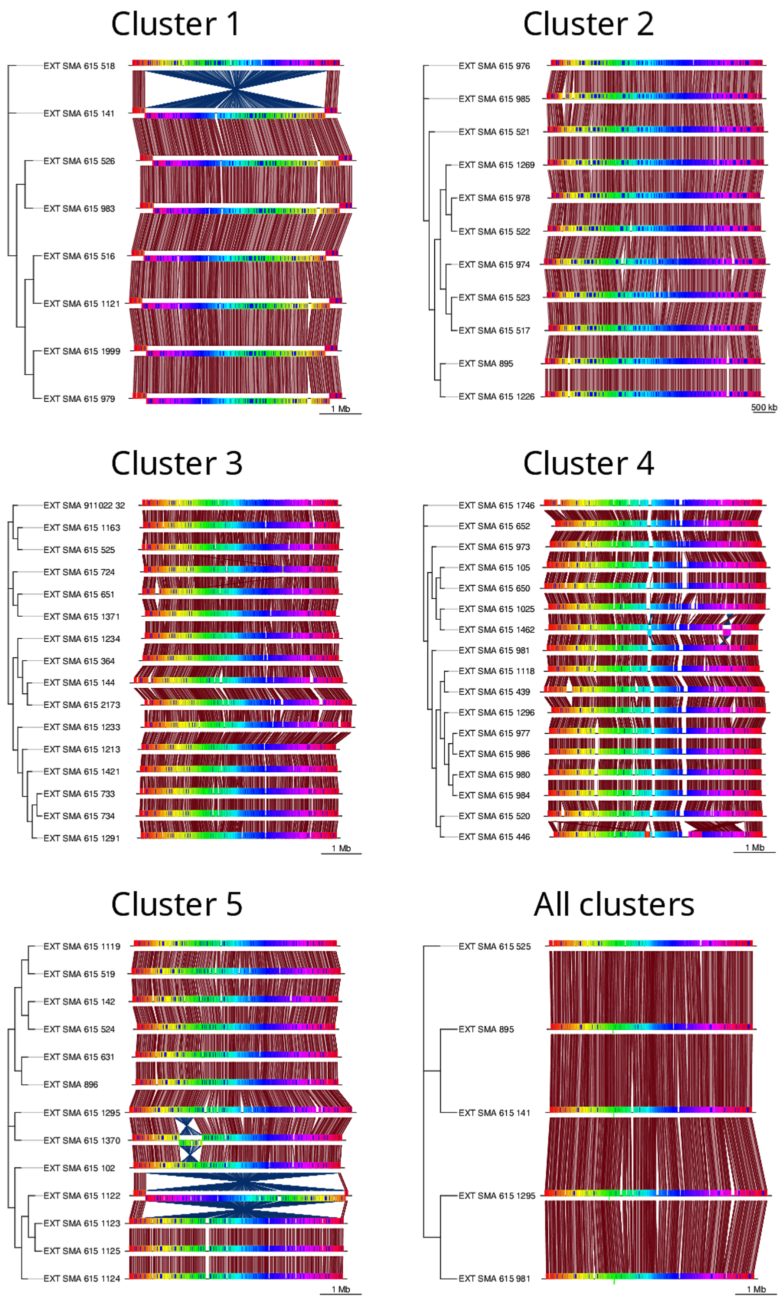

Figure S4. Synteny between *S.marcescens* clusters.

Visualisation of the synteny between the 65 complete genomes present in the study. The synteny is shown for strains within each cluster. On the bottom right ("All clusters"), the synteny between clusters is visualised using one genome representative of the most common syntenic variant for each cluster.

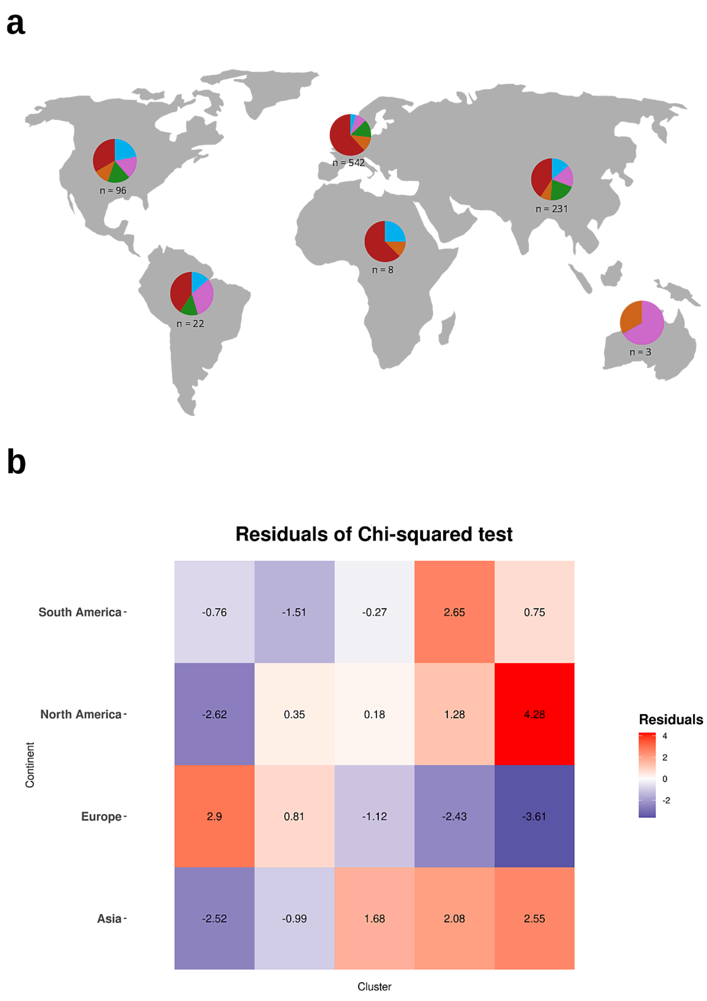

Figure S5. Macrogeographical distribution of *S.marcescens* clusters.

a) Pie charts representing the frequency of each cluster in the six continents (starting from the left: on top North America, Europe, Asia; at the bottom South America, Africa, Oceania). The total number of strains isolated in the continent (n) is shown under each pie chart. (The world map used for this image has been downloaded from website https://freesvg.org/) b) Chi-squared test was used to assess whether *S. marcescens* clusters are associated with geographical continents. The heatmap shows the Pearson residuals of the Chi-squared test and statistically significant associations are marked with an asterisk (*).

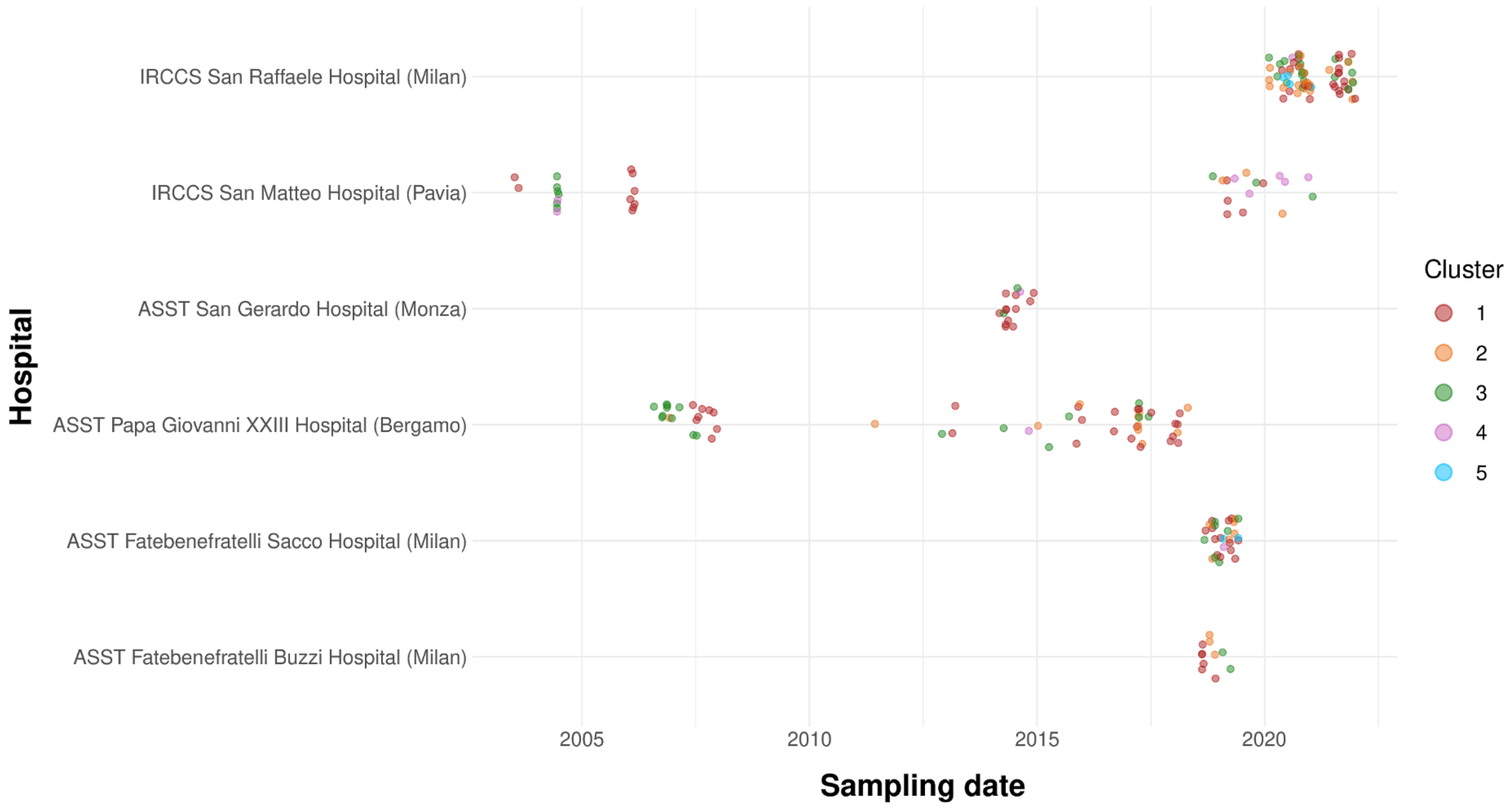

Figure S6. Spatiotemporal distribution of *S. marcescens* in Italian hospitals.

Plot showing the spatiotemporal distribution of 235 *S.marcescens* strains sampled from six Italian hospitals.

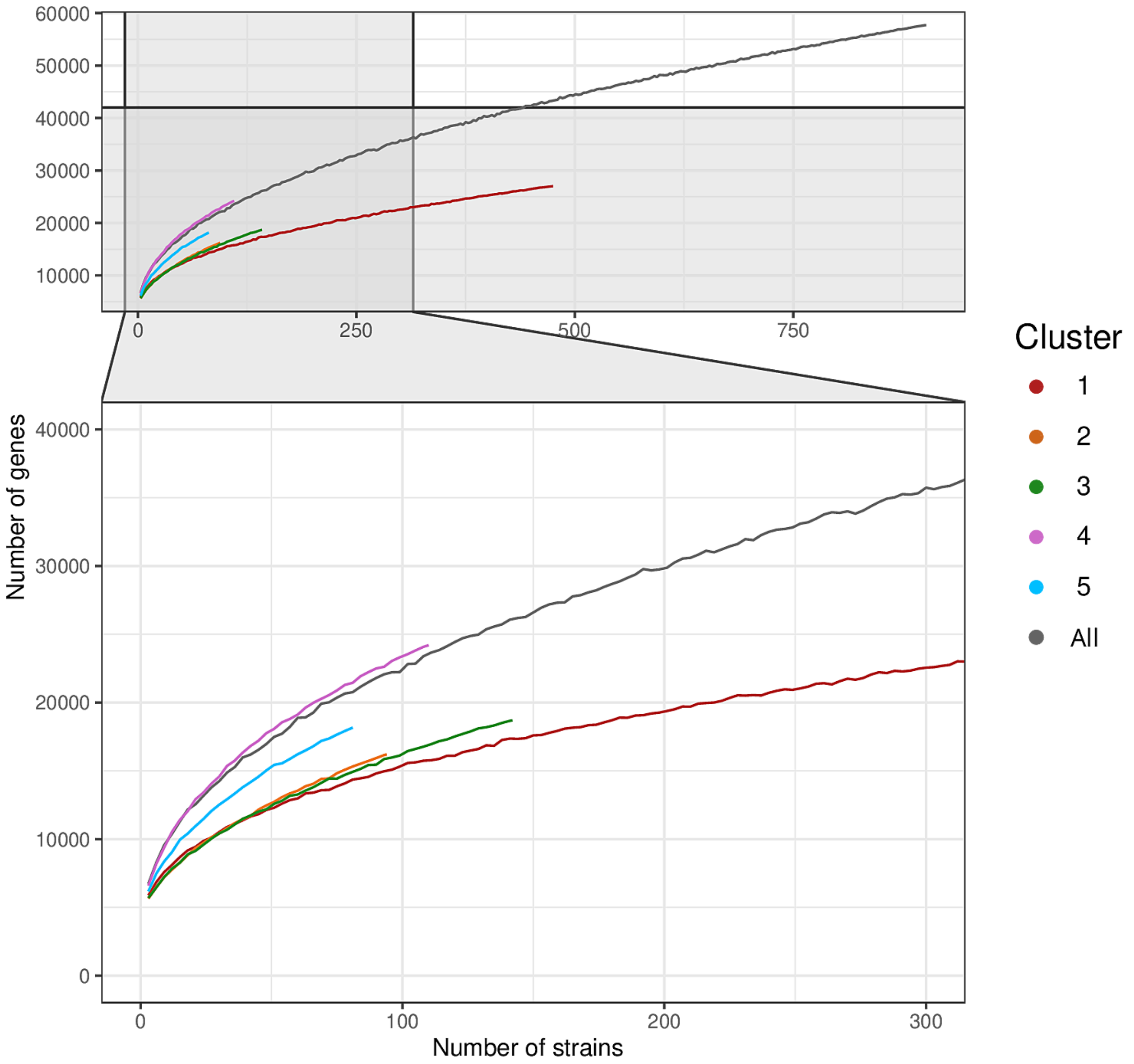

Figure S7. Pan-genome curves for *S.marcescens* clusters.

Pan-genome accumulation curves for each cluster of *S. marcescens* strains. The region between 1 and 300 strains is zoomed to allow a better visualisation of curves belonging to smaller clusters. Clusters are coloured according to the key on the right.

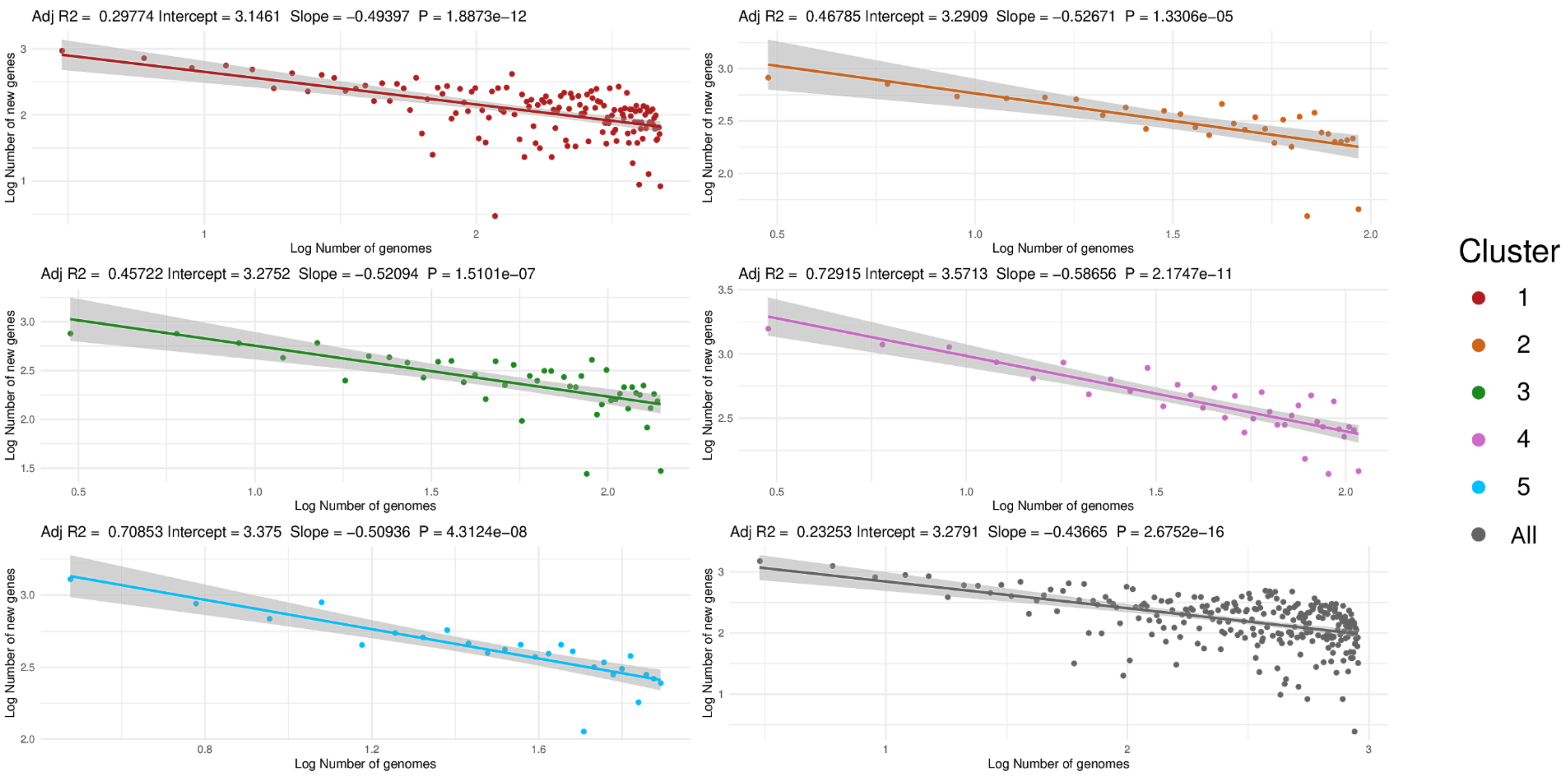

Figure S8. Analysis of the pan-genome openness for *S.marcescens* clusters.

The plot shows the log-log regression for new genes found in an increasing number of genome sequences within *S.marcescens* clusters. A slope of the regression line < 1 indicates that when more genomes are analysed, the number of new genes decays slowly, thus the pan-genome is open.

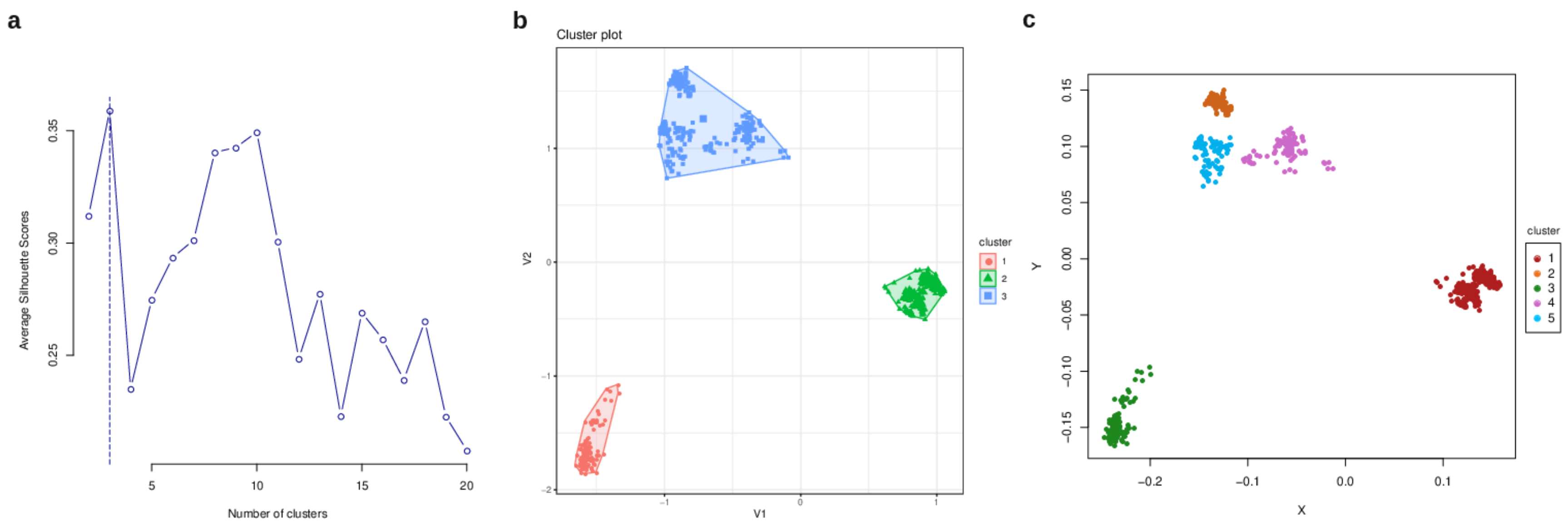

Figure S9. K-means clustering on gene presence absence-based Jaccard distances.

a) Silhouette plot of the number of clusters on the basis of Jaccard distances computed from gene presence absence between *S. marcescens* strains. The number of clusters with the highest score was chosen as the optimal number of clusters to fit the data in. b) Principal Coordinate Analysis (PCoA) performed on Jaccard distances computed from gene presence absence, coloured on the basis of the clusters identified by the K-means algorithm. c) Principal Coordinate Analysis (PCoA) performed on Jaccard distances computed from gene presence absence, coloured on the basis of the five phylogenetic clusters identified in the *S. marcescens* population.

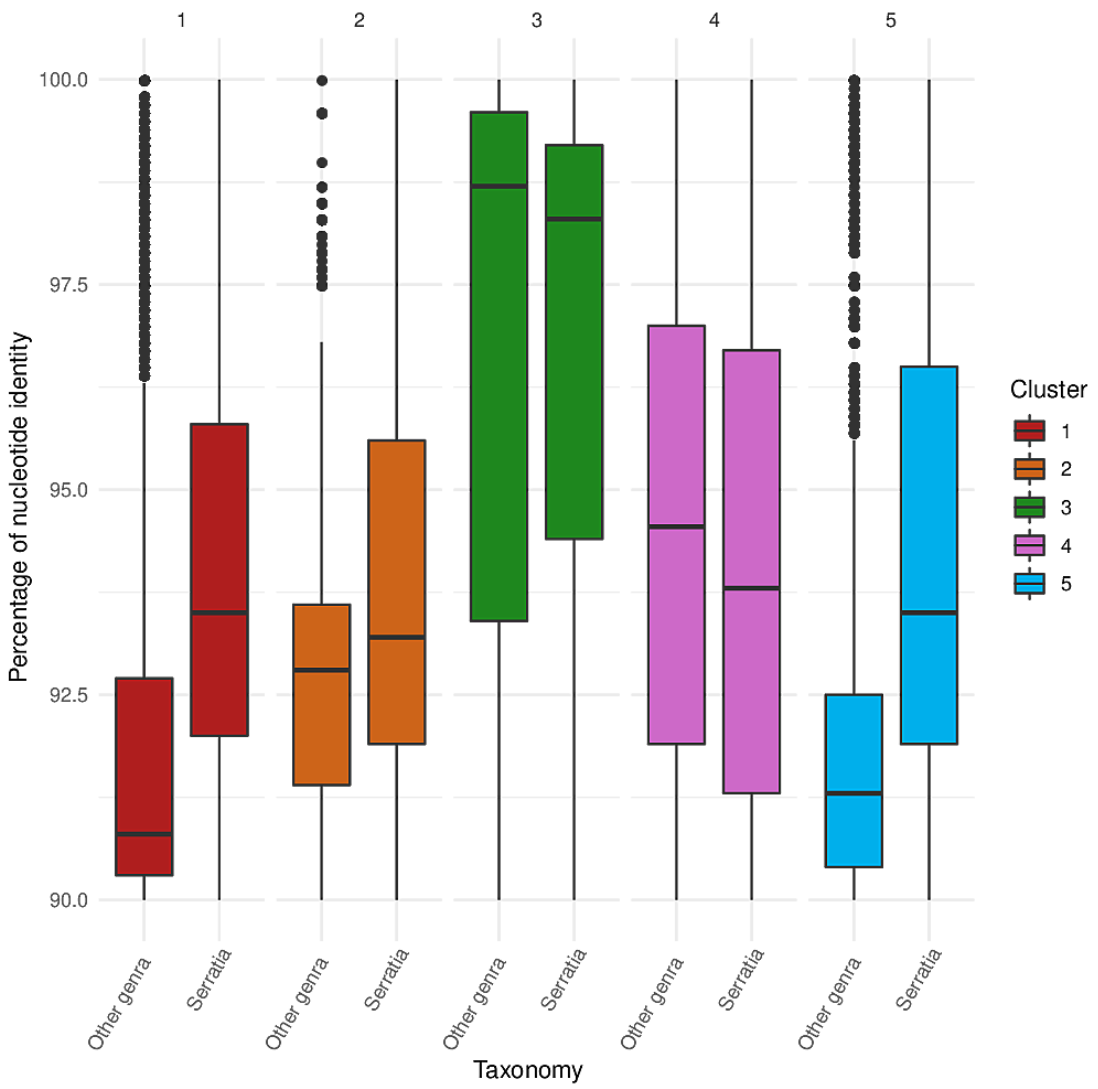

Figure S10. Sequence similarity between proteins of cluster-specific core genes and sequences outside *S. marcescens.*

Boxplot showing the percentages of nucleotide identity obtained comparing protein sequences of cluster-specific core genes against protein sequences not belonging to *Serratia marcescens* on the NCBI nr database.

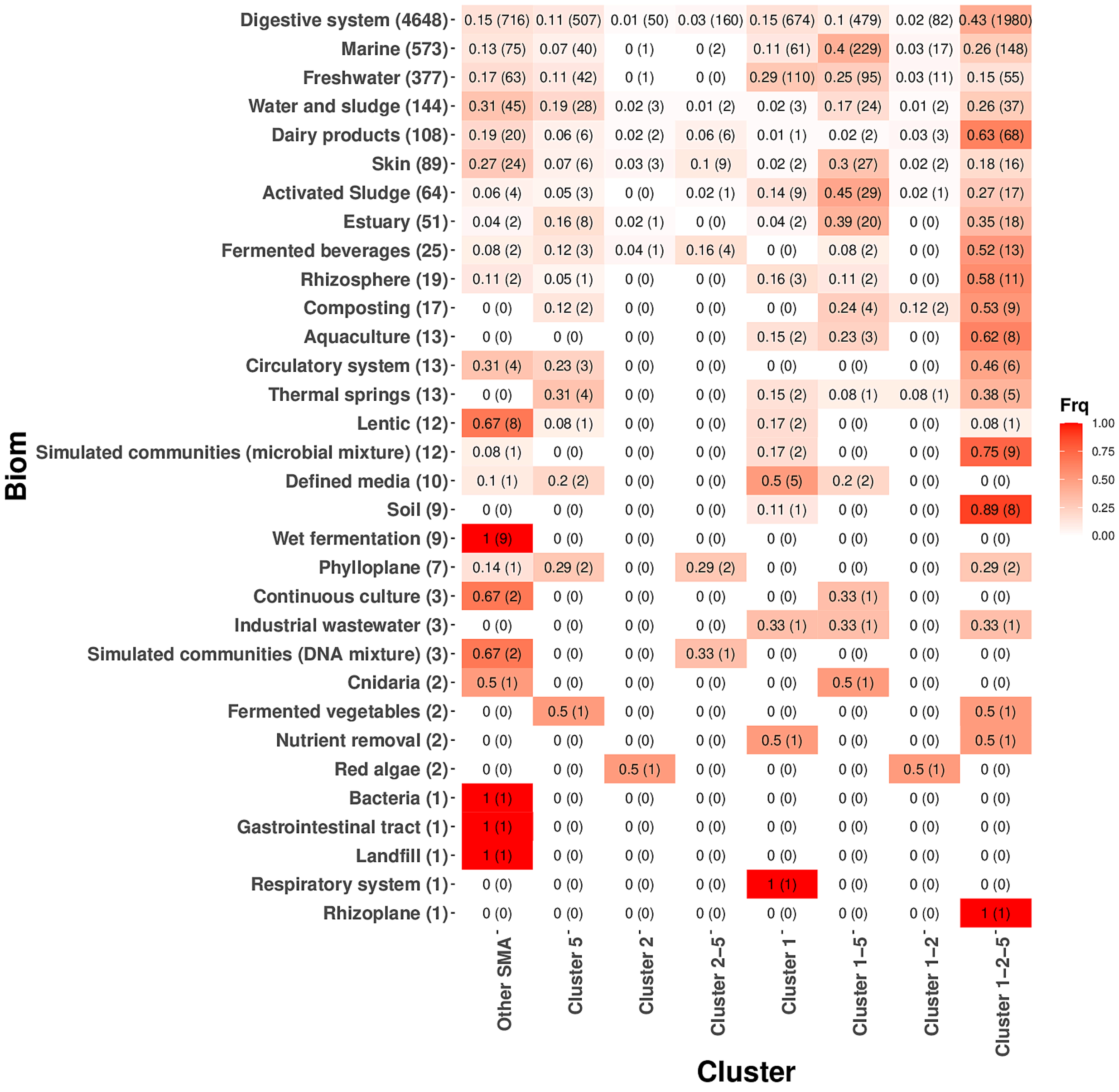

Figure S11. Presence of *S. marcescens* clusters in metagenomic samples from different ecological sources/biomes

The presence of Cluster 1, Cluster 2, Cluster 5 and other S. *marcescens* was investigated in metagenomic samples of the MGnify database searching for cluster-specific core genes and *S. marcescens* core genes. The heatmap shows the frequency with which every combination of clusters was found in samples from different ecological sources/biomes. In brackets, the number of samples for each combination.

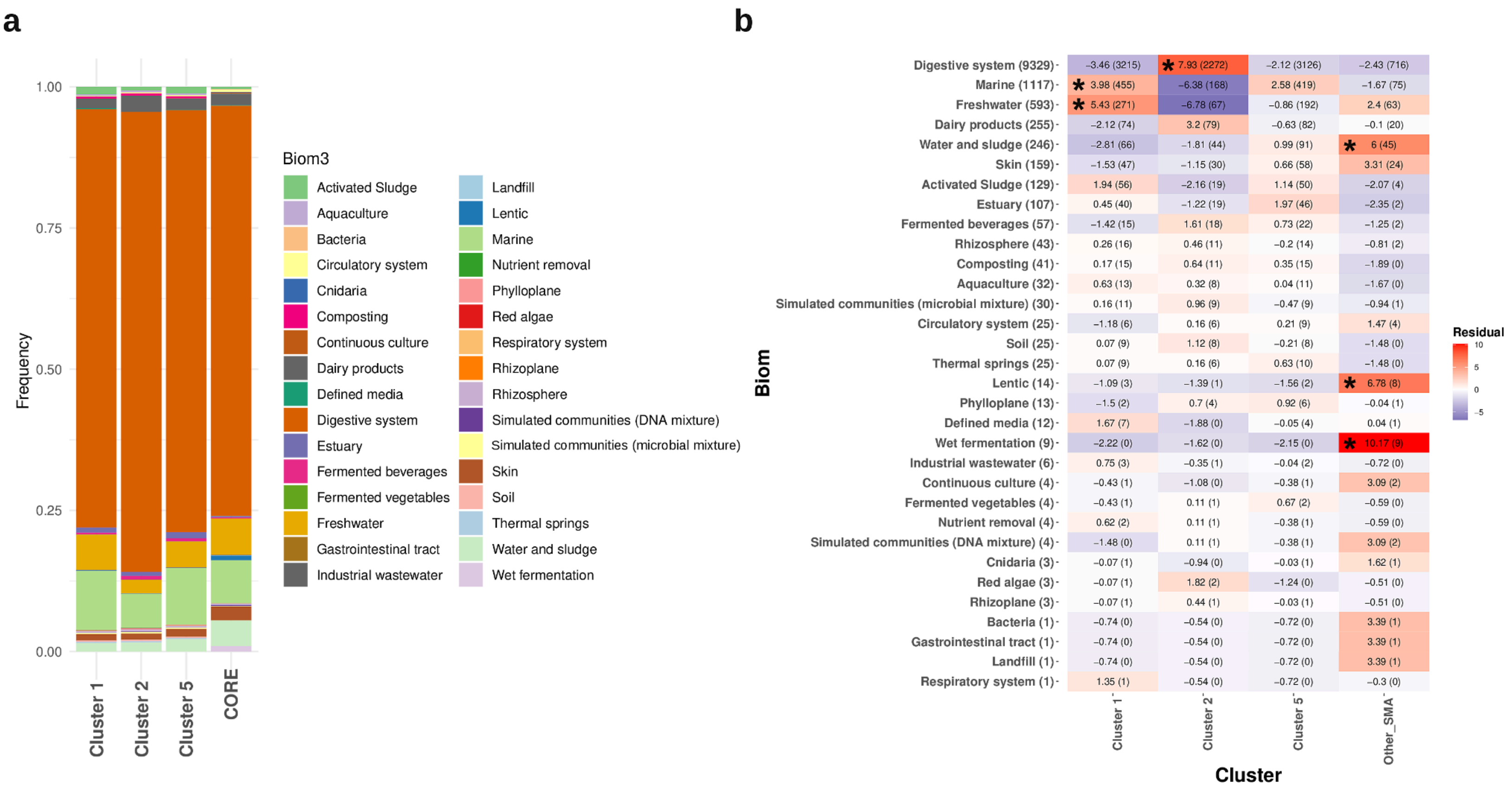

Figure S12. Inference of *S. marcescens* clusters in metagenomic samples

a) The stacked bar chart shows the biomes of metagenomic assemblies from the MGnify database in which *S. marcescens* clusters were identified. An assembly was considered positive to a Cluster if >10% of cluster-specific core genes were found within it. When a metagenomic assembly resulted positive to core *S. marcescens* genes, but not to genes associated to Cluster 1, Cluster 2 and Cluster 5, the assembly was defined as “Other SMA”. b) The heatmap shows the residuals of the Chi-squared test used to investigate whether *S. marcescens* clusters are associated to samples from specific biomes. Statistically significant associations are marked with asterisks (‘*’, p value <0.05).

a

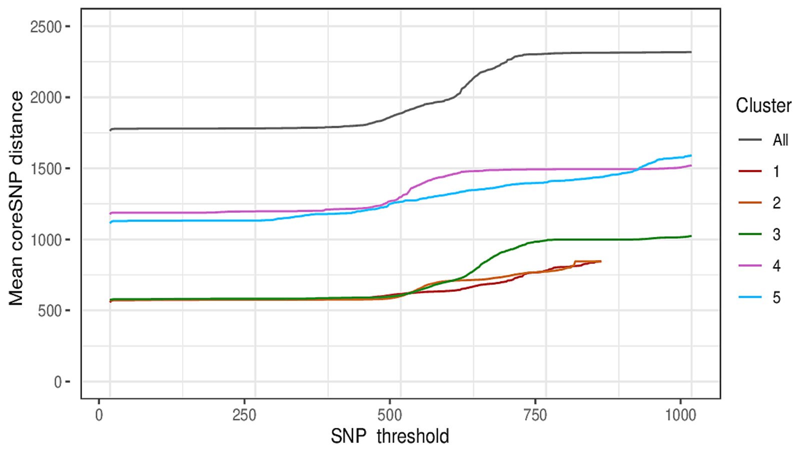

b

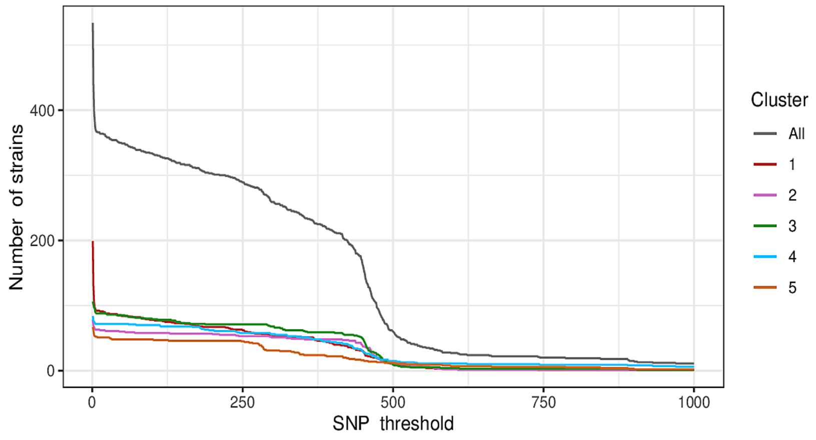

Figure S13. Determination of the optimal coreSNP threshold to create the Refined genomic dataset

In order to reduce the size of the dataset and the genetic bias (for recombination, gene flow and molecular clock analyses) the 902 *S. marcescens* genomes were grouped in coreSNP groups on the basis of a threshold in pairwise coreSNP distance. The determination of the threshold was optimised to reduce and balance the dataset as much as possible with a minor impact on the represented genetic variability. a) To evaluate the potential loss of genetic variability, the mean coreSNP distance for each cluster between strains was plotted for every coreSNP threshold between 0 and 1000 b) To evaluate the size reduction of the dataset, the number of coreSNP groups was plotted for every coreSNP threshold between 0 and 1000. On the basis of both plots, a threshold of 500 coreSNPs was considered optimal.

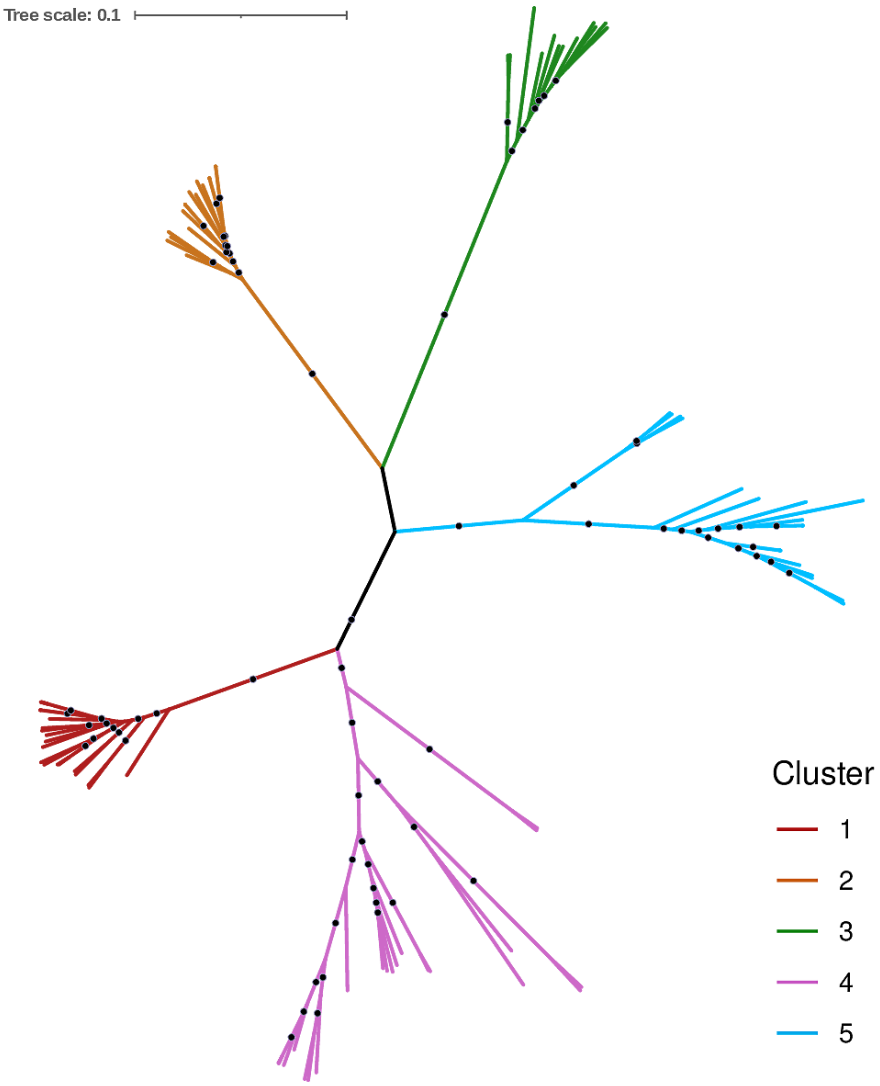

Figure S14. Unrooted phylogenetic tree of *S. marcescens* strains in the Refined genomic dataset

SNP-based Maximum Likelihood (ML) phylogenetic tree (performed with 100 pseudo-bootstrap) including the 86 *S. marcescens* strains of the Refined genomic dataset. The five clusters correspond to five monophyletic groups. Bootstraps above 90 are represented as black dots on the corresponding node.

a

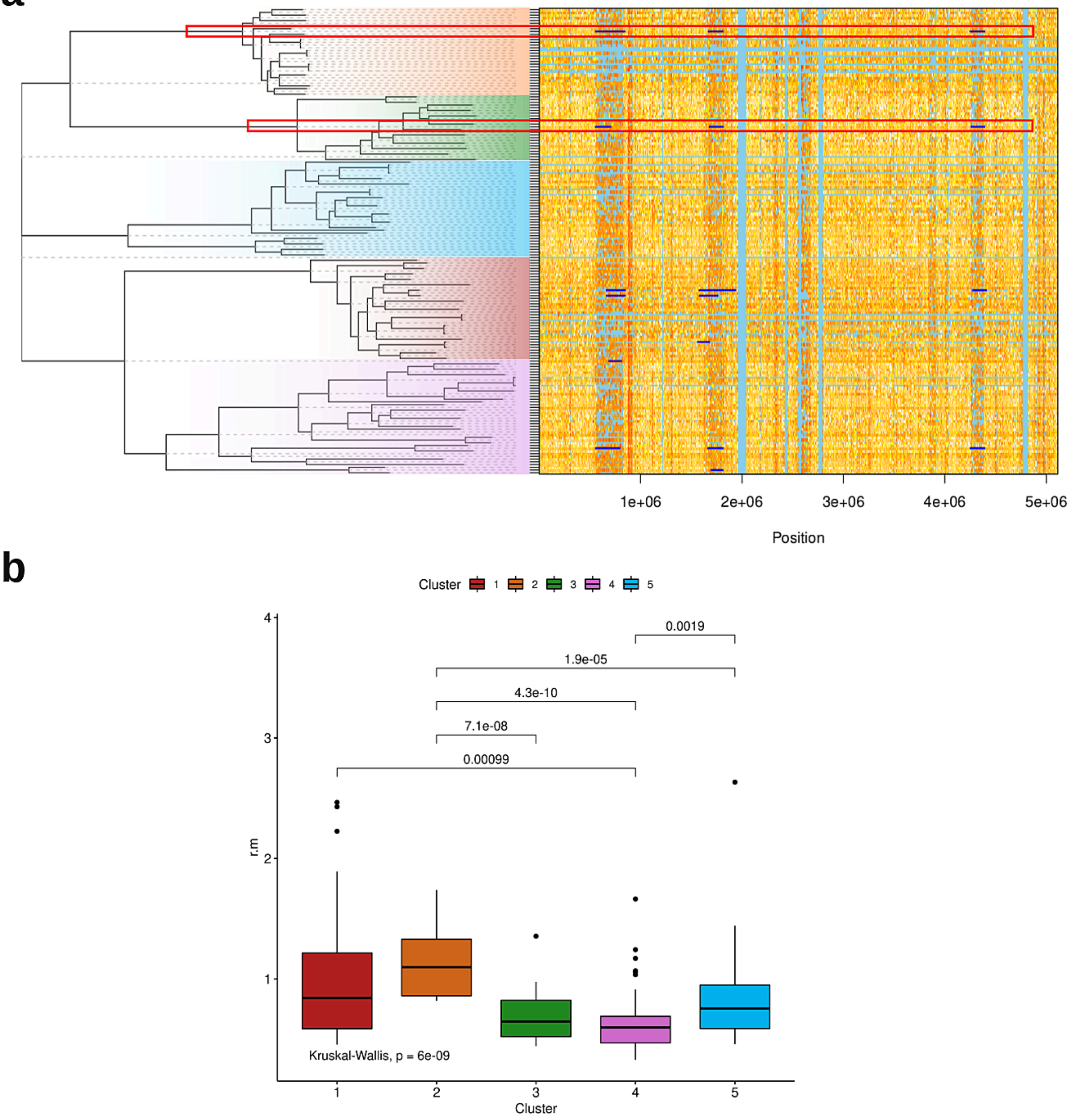
1e+06
2e+06
3e+06
4e+06
5e+06
Position
b
Cluster 1 2 3 4 5
r.m
0.0019
1.9e-05
4.3e-10
7.1e-08
0.00099
Kruskal-Wallis, p = 6e-09
Cluster

Figure S15. Large recombinations and recombination to mutation ratio in *S. marcescens* clusters

a) Plot illustrating large recombination events (>50 kbp) along the genome (on the right) mapped on every node of the phylogenetic tree (on the left). The colours on the phylogenetic tree represent the *S. marcescens* clusters and the dashed red lines indicate the basal node of each cluster. For Cluster 4, the basal node does not correspond to any large recombination. b) Boxplot showing the recombination to mutation ratio (r/m) for the nodes of each cluster. The p-value of the Kruskal-Wallis test, performed to test the variance between groups, is shown on the bottom left. The groups with significant pairwise differences (Mann-Whitney U test) are connected and the corresponding p-value is written on top.

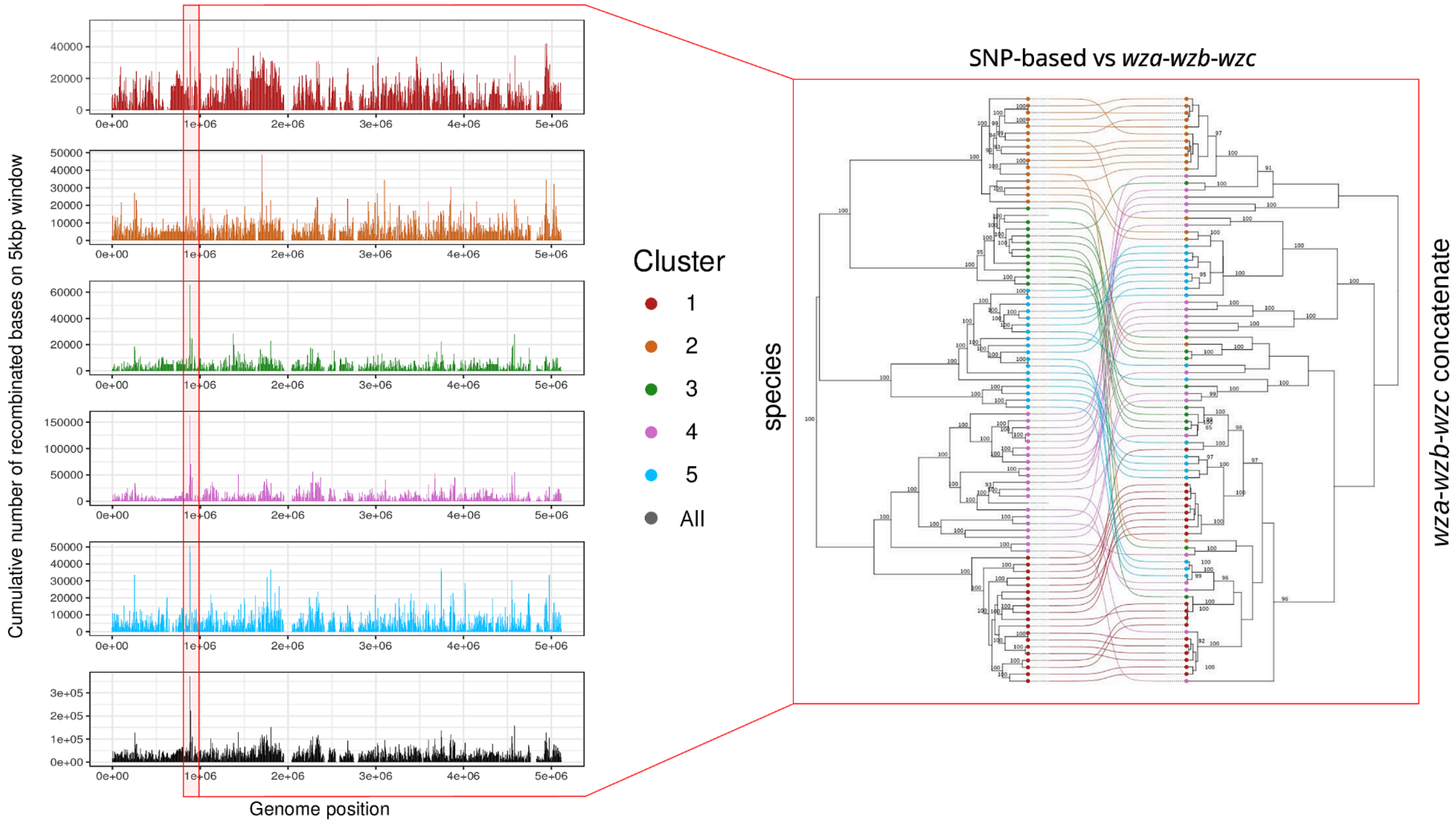

Cumulative number of recombinated bases on 5kbp window
Genome position
Cluster
1
2
3
4
5
All
SNP-based vs *wza-wzb-wzc*
species
wza-wzb-wzc concatenate

Figure S16. Recombinations along the *Serratia marcescens* genome in each cluster

On the left, distribution of cumulative recombination events along the *Serratia marcescens* genome. The analysis has been performed on a multi-genomes alignment of 86 strains selected to be representative of the clusters (genome alignment was obtained using the genome of *S. marcescens* Db11 strain as reference, position on the x-axis refer to this genome assembly). For each cluster, the recombination distribution is shown as the cumulative number of recombinated bases for genomic windows of 5 kbp. A 10-kbp long highly-recombinated region, containing the capsular genes *wza*, *wzb* and *wzc*, is highlighted by the red box. On the right, comparison among the Maximum Likelihood (ML) phylogenetic trees of the 86 strains, obtained using coreSNPs (on the left) and *wza*-*wzb*-*wzc* concatenate (on the right). The results highlight that capsular genes are recombination hotspots.

Supplementary Note 1. Statistically significant P values in the comparison of genome size and GC content between *S. marcescens* clusters

Genome size and GC content between strains of different clusters were compared by Mann-Whitney U-test with Holm post-hoc correction.

Genome size: Cluster 4 has a wider genome size in comparison to Cluster 1 ($p = 1.8e-05$, Mann-Whitney U test), Cluster 2 ($p = 1.4e-07$), Cluster 3 ($p = 2.5e-10$) and Cluster 5 ($p = 2.2e-06$). Cluster 1 genomes are also significantly larger than genomes in Cluster 3 ($p = 0.00013$, Mann-Whitney U test).

GC content: Cluster 1 has a markedly higher GC content than Cluster 2 ($p < 2.22e-16$, Mann-Whitney U test), Cluster 3 ($p < 2.22e-16$), Cluster 4 ($p < 2.22e-16$) and Cluster 5 ($p = 3.8e-14$). At the same time, Cluster 2 also has a lower GC content than Cluster 3 ($p < 2.22e-16$)
