# Supplementary material for: Genetic barriers more than environmental associations explain Serratia marcescens population structure
Source: Commun Biol. 2024 Apr 17;7:468. doi: 10.1038/s42003-024-06069-w (PMC11023947; doi:10.1038/s42003-024-06069-w)
Supplement: Supplementary file 2 — Description of Additional Supplementary Files [file 42003_2024_6069_MOESM2_ESM.pdf]

Description of Additional Supplementary Files

File name: Supplementary Data 1
Description: Accession numbers and metadata of *S. marcescens* strains used in the study.

File name: Supplementary Data 2
Description: COG annotation of *S. marcescens* cluster-specific core orthologous groups.

File name: Supplementary Data 3
Description: Annotated R-M systems found in *S. marcescens* clusters.
